# What Are the Self-Assessed Training Needs of Early Career Professionals in Addiction Medicine? A BEME Focused Review

**DOI:** 10.15694/mep.2020.000062.1

**Published:** 2020-04-03

**Authors:** Jan Klimas, Damien Kelly, Ahmed Adam, Sidharth Arya, Blanca Iciar Indave Ruiz, Dzmitry Krupchanka, Michee-Ana Hamilton, Thomas Dennehy, Evan Wood, Walter Cullen

**Affiliations:** 1 University College Dublin; 2 British Columbia Centre on Substance Use; 3 Institute of Mental Health; 4 Instituto de Salud Carlos III; 5 World Health Organization

**Keywords:** Substance-related disorders, addiction medicine, training needs, health personnel, educational/training needs, continuing medical education

## Abstract

This article was migrated. The article was marked as recommended.

**Background:** Substance use disorders represent a significant social and economic burden globally. Accurate diagnosis and treatment by early career professionals in addiction medicine (ECPAM) falls short, in part, due to a lack of training programmes targeting this career stage. Prior research has highlighted the need to assess the specific training needs of ECPAM. Therefore, this focused review assessed self-reported training needs of ECPAM.

**Methods:** Medical and medical education databases (Medline, EMBASE, CINAHL, ERIC, PSYCHInfo, BEI, and AEI) were searched to June 2018 for studies reporting self-reported training needs of ECPAM (trained at most five years before assessment occurred). Retrieved citations were screened for eligibility; two independent researchers reviewed included studies, assessed quality and extracted data. Experts reviewed study findings.

**Results:** Of 1364 identified records, three cross-sectional studies were included, originating from China, USA and England. All studies surveyed ECPAM using self-reported questionnaires, with one study including face-to-face interviews. Participants included residents, physicians and social workers. All studies had a low risk of bias, and reported a wide range of training needs including rehabilitation, relapse prevention, buprenorphine treatment and risk assessment.

**Conclusions:** There is little evidence for and substantial heterogeneity of training needs of ECPAM found in this review, particularly at the level of skills and knowledge. Study quality varies greatly. ECPAM training needs assessments are a priority.

## Background

Substance use disorders (SUDs) represent a significant social and economic burden globally ( Degenhardt *et al.*, 2018), with the number of people affected continuing to rise ( United Nations Office on Drugs and Crime, 2018), according to the data from Global Burden Of Disease Study 2016 ( Degenhardt *et al.*, 2018). Overall, SUD-related physical and psychological morbidity put challenging demands on healthcare systems ( Murray *et al.*, 2013). Advances in addiction science have helped to improve care for people with SUDs, aiding in early identification and treatment ( Kaner *et al.*, 2009). In addition, health care professionals contribute significantly to improved early management of SUDs, as has been described by numerous reports ( Cullen *et al.*, 2006; Kaner *et al.*, 2013).

Recently, the formal recognition of addiction medicine by the American Board of Medical Subspecialties, as a multispecialty subspecialty in 2016, and the implications for professionals in this discipline, have been described in North American settings ( The American Society of Addiction Medicine, 2009; Wood, Samet and Volkow, 2013; O’Connor, Sokol and D’Onofrio, 2014). The new discipline’s main educational pathway, an addiction medicine fellowship, started with training early-career physicians but recently expanded to other disciplines, such as social work, pharmacy and nursing ( The American Society of Addiction Medicine, 2009; Wood, Samet and Volkow, 2013; O’Connor, Sokol and D’Onofrio, 2014). In 2011, the first 9 fellowship programs were accredited by the American Board of the Addiction Medicine Foundation that has been renamed to Addiction Medicine Foundation in 2015, and again in 2019 to its current name, the American College of Academic Addiction Medicine, with a total of 62 programs. The fellowship programs provide 12-month structured curriculum to fellows regardless of their specialty.

Early career is a crucial stage in the development of health professionals. However, adequate diagnosis and treatment of SUDs by early career professionals in addiction medicine (ECPAM) falls short ( Degenhardt *et al.*, 2017), in part, due to a lack of treatment coverage, qualified workforce and accredited early-career training programmes ( The National Center on Addiction and Substance Abuse at Columbia University, 2000; Ries *et al.*, 2009; The National Centre on Addiction and Substance Abuse at Columbia University, 2012). In North America, new educational pathways, including medical fellowships to train ECPAM in the diagnosis and treatment of SUDs, are emerging ( El‐Guebaly *et al.*, 2002). However, the World Health Organization data suggest that other countries may not have such options and the specific training needs of these individuals remain unclear ( World Health Organization, 2017c; World Health Organization, 2017a).

Prior research has highlighted the need for exploration of the specific training needs of healthcare professionals in the area of addiction medicine ( Ayu *et al.*, 2017). For example, a recent review found that while the need for improved education in addiction medicine is internationally recognised, there remains a stark imbalance in the implementation of established addiction medicine curricula between western and non-western nations ( Ayu *et al.*, 2015).

In light of both the pressing need for improved training in addiction medicine, and a lack of research from different educational settings, this focused review sought to identify self-reported training needs of ECPAMs by carrying out systematic searches of online medical and educational databases.

## Methods

This review followed a published protocol that has been reviewed by the Best Evidence Medical Education (BEME) Collaboration. BEME’s standards and guiding in research have been outlined in detail previously ( Harden *et al.*, 1999). A search of several medical literature databases (MEDLINE, EMBASE, CINAHL, etc.) to June 2018 was performed, with no limitation on publication date, for studies reporting self-perceived training needs of early career professionals (having completed their training within a five-year period at the time of the study) in addiction medicine. Medical Subject Headings (MeSH) terms and key words relating to substance-related disorders; addiction medicine; training needs; health personnel were used (see Supplementary file 1). Two authors (DK and AA) independently screened the titles and abstracts retrieved from the search for inclusion. The full texts of all potentially eligible studies were obtained. DK and AA then independently assessed the full texts against the eligibility criteria for inclusion, with JK acting as an arbitrator where there was disagreement. In addition, reference lists from all included studies were consulted for further relevant publications.

To be eligible for inclusion in the review, the study must have surveyed early career professionals (operationalised as any professional who has completed their training within a 5-year period at the time of data collection) who provide services or care to patients with substance-use disorders. The study also must have included self-reported training needs of the ECPAM, as measured by standardised training needs assessments (TNA), other questionnaires, interviews, focus groups, surveys or any other self-report measure aimed at identifying the training needs of ECPAM. We used Ratnapalan and Hilliard’s definition of ‘perceived needs,’ i.e., what the individuals or the group have identified as what they want to learn ( Ratnapalan and Hilliard, 2009). Articles that met any of the following criteria were excluded from the review: a) studies that did not have training needs assessments in substance use care as an outcome; b) non-English language studies; c) studies focused exclusively on undergraduate students; d) studies focused exclusively on specialists or those who completed their training beyond a five-year period; e) studies focused exclusively on evaluating an educational intervention, and/or f) studies focused exclusively on ECPAM curriculum content. Due to the sparse literature, the planned review was changed to a focused review ( Gordon *et al.*, 2019).

Data was extracted by two independent reviewers experienced in literature reviewing (DK and AA) using the process of Charting ( Ritchie and Spencer, 1994; Gordon *et al.*, 2019), including: study design, setting, sample, methodology, outcome measures, previous training/experience and training needs identified. As per the published protocol, this review’s primary outcomes of interest were self-reported training needs of ECPAMs working internationally. The Joanna Brigg’s Institute (JBI) Checklist for Analytical Cross Sectional Studies assessed the risk of bias (Supplementary file 1) ( Moola *et al.*, 2017). A thematic synthesis summarised the extracted data. The protocol sought to categorise the training needs under representative themes, organised in a table containing the number and percentage of studies and/or participants reporting needs of each theme. This review was conducted as a part of Training Needs in Addiction Medicine collaborative project implemented by the Network of Early Career Professionals working in the area of Addiction Medicine (NECPAM), under the auspices and with support from Management of Substance Abuse Unit, Department of Mental Health and Substance Abuse, World Health Organization (WHO). Experts identified by NECPAM reviewed the study findings to check if we missed any important literature sources, misinterpreted existing data sources as well as validated the key messages of the paper. In particular, we collected feedback from members of NECPAM and experts in the field of addiction medicine training by attending select international scientific meetings (WHO Forum on Alcohol Drugs and Addictive Behaviours 2017, International Society of Addiction Medicine 2017, Lisbon addictions 2017, World Psychiatry Association 2018, European Psychiatric Association 2018, International Conference at Ain Shams University in Cairo 2018, International Society for Biomedical Research on Alcoholism 2018, South African Addiction Medicine Society and Africa and Middle East Congress on Addiction 2018, and Congress of the European Society for Social Psychiatry 2018). The situational analysis of training needs of ECPAM and workforce development in addiction medicine was presented by representatives of countries from various world regions, followed by extensive discussions with participants during and after the above-mentioned events. As mentioned above, preliminary results were presented at several occasions with inquiries related to (1) correctness of review conclusions; (2) additional references missed by the review; (3) experiences of training in other countries. In the process of these consultations, no objections were received with regards to the key findings and conclusions of the review. Only a few additional references have been suggested, as well as a range of ideas collected on next steps that could be undertaken to strengthen training (e.g., on training elements missing in terms of delivery and content).

## Results

### Focused review results

**Figure 1.**
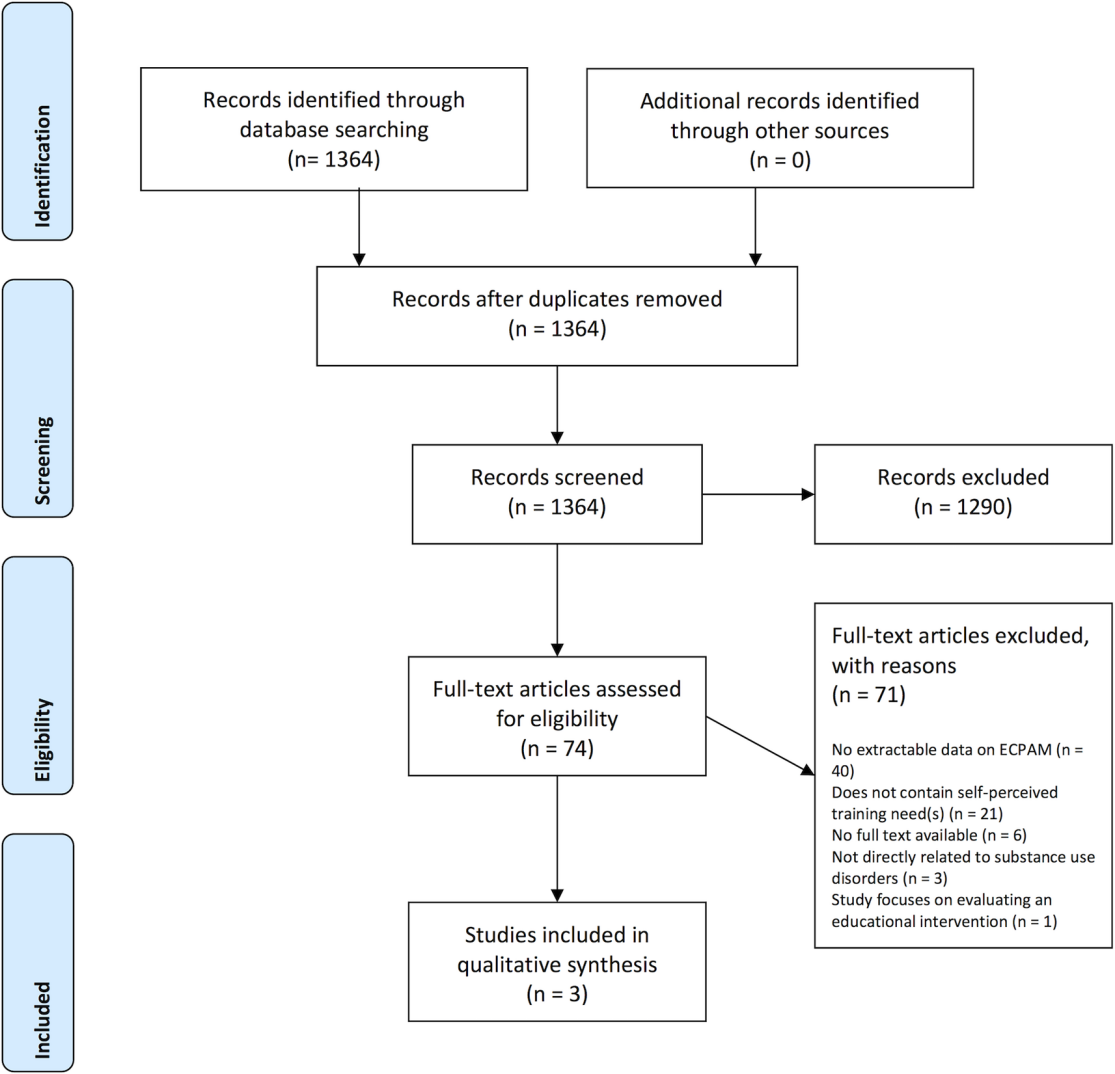
Study flow chart. Self-reported training needs of ECPAM Preferred Reporting Items for Systematic Reviews and Meta-Analyses (PRISMA) flow diagram.

After de-duplication, a total of 1364 unique citations and abstracts were retrieved from the initial search. A total of 1290 records were excluded at the abstract screening stage as they did not meet the eligibility criteria. The number of full texts assessed for eligibility was 74. Of those, 71 articles were excluded for the following reasons: there was no extractable data on ECPAM (n=40); the study did not report self-perceived training need(s) (n=21) ( Figure 1). Additionally, six studies were excluded as the full text was not available, three did not focus directly on substance-use disorders and one focused on evaluating an educational intervention. Three studies were included and analysed thematically ( Tang *et al.*, 2005; Cunningham *et al.*, 2006; Galvani and Forrester, 2011).

PRISMA Flow Diagram. Creative Commons Attribution License 4.0. Adapted with permission from Moher, D., Liberati, A., Tetzlaff, J., Altman, D. G., *et al*. (2009) ‘Preferred Reporting Items for Systematic Reviews and Meta-Analyses: The PRISMA Statement’, PLoS Medicine 6(7). https://doi.org/10.1371/journal.pmed.1000097

### Characteristics of included studies

The characteristics of the included studies are detailed in Table 1. All of the studies had a cross-sectional design, involving 793 early career professionals in addiction medicine (physicians of various specialisations 58.9%, residents 2.6% and social workers 31.2%) from China, England and the U.S. ( Tang *et al.*, 2005; Cunningham *et al.*, 2006; Galvani and Forrester, 2011). All studies used a self-reported questionnaire and one of the studies also used a face-to-face interview. Questionnaires that were used in the included studies were not assessed for reliability and validity, except one that was a modified version of a tool used in a previous study ( Cunningham *et al.*, 2006). Identification of training needs was the primary outcome of only one article ( Galvani and Forrester, 2011). For the other two studies, the primary outcomes were assessing the beliefs and opinions of Chinese health care professionals toward substance use and treatment ( Tang *et al.*, 2005); and assessing the attitudes and beliefs of primary care physicians towards substance use treatments with buprenorphine ( Cunningham *et al.*, 2006). Quality appraisal scores are shown in Table 1. All included studies were determined to have low risk of bias, although none of them used objective, standard criteria for the measurement of the condition in the form of validated questionnaires.

### Training needs of early career professionals in addiction medicine

While the training needs identified in the three included studies were heterogeneous, the *treatment* and *management* of substance use disorders emerged as a common theme across studies ( Table 1). Only a few different health care professions were included in the reviewed studies.

Cunningham *et al*., surveyed 78 residents (80.4%) and 21 attending physicians (19.6%) in a cross-sectional survey, via questionnaire and face-to-face interview ( *et al.*, 2006). The attitudes and beliefs of primary care physicians towards opioid use disorder (OUD) treatment, particularly buprenorphine, were assessed. The study reported the most common training and support needs, including education and training specific to buprenorphine (83.8%), and whether further training would influence buprenorphine prescribing habits. A statistically significant difference for willingness to prescribe buprenorphine with proper training was identified between primary and non-primary care physicians (81.3% versus 45.5%, P<.05), potentially indicating a greater self-assessed training need. While this study did not use standard criteria to measure the condition, overall the risk of bias was low.

Galvani and Forrester e-surveyed 284 newly qualified social workers (NQSW), and examined their self-assessed preparedness for working in alcohol and drug use settings ( Galvani and Forrester, 2011). The NQSW rated how adequately their social work programmes prepared them for front line work with alcohol and drug use. Training needs identified in the study emerged based on insufficient content available in each respondent’s social work programme. The addiction medicine training needs self-identified by the respondents ranked in the following order (from most to least important):

1. How to assess risk relating to drug or alcohol issues (74%).
2. Substance use and issues of ethnicity and culture (73%)
3. Types of intervention/treatment available (71%)
4. Impact on mental health (67%)
5. How to talk about drug or alcohol issues (66%)
6. Gender differences (65%)
7. Working with specialist substance use colleagues/referrals (62%)
8. Identifying problematic drug use (61%)
9. Impact on children and families and parenting (60%)
10. Identifying problematic alcohol use (59%)
11. Impact on physical health (54%)
12. Drugs and their effects (50%)
13. Alcohol and its effects (47%)
14. Attitudes and values relating to substance use and problems (47%)
15. Reasons people use (41%)

Tang *et al*., surveyed (Tang *et al.*, 2005) 446 respondents working in voluntary (57.4%) or involuntary (42.6%) Drug Abuse Treatment Facilities (DATFs) using a questionnaire. The self-assessed training needs were assessed as a secondary outcome. More than 52% of all respondents desired training in rehabilitation and relapse prevention. The second most important need was detoxification-related knowledge (37.9%). The third most important need was aftercare and related topics (9.6%). The only identified training need that was significantly different between involuntary (28.4%) and voluntary (44.9%) DATFs workers was need for detoxification-related training (p < 0.01). Nevertheless, there was a lack of clear inclusion criteria for the sample, indicating potential selection bias. In sum, training specific to buprenorphine, risk-assessment, rehabilitation and relapse prevention appeared as the most important self-assessed training needs.

**Table 1.** Characteristics of included studies.

Author, Year	Country	Design	Participants	Outcome Assessed	Training Needs Identified	Quality
**Cunningham *et al*., 2006**	**USA**	**Cross-sectional survey with questionnaire and interview**	**n = 78 residents (80.4%);n = 21 attending physicians (19.6%)**	**1. Attitudes and beliefs towards treatments for opioid addiction, including buprenorphine** **2. Association between opioid training and attitudes toward treating substance users**	**1. Education and training specific to buprenorphine (83.8%)**	7/8*
**Galvani and Forrester, 2011**	**England**	**Cross-sectional survey with questionnaire**	**n = 248 social workers**	**1. Perceived preparation for social work practice with an emphasis on substance use,** **2. Substance use training post-qualifying and future training needs.**	**1. How to assess risk relating to drug or alcohol issues (74%)** **2. Substance use and issues of ethnicity and culture (73%)** **3. Types of intervention/treatment available (71%)** **4. Impact on mental health (67%)** **5. How to talk about drug or alcohol issues (66%)** **6. Gender differences (65%)** **7. Working with specialist substance use colleagues/referrals (62%)** **8. Identifying problematic drug use (61%)** **9. Impact on children and families and parenting (60%)** **10. Identifying problematic alcohol use (59%)** **11. Impact on physical health (54%)** **12. Drugs and their effects (50%)** **13. Alcohol and its effects (47%)** **14. Attitudes and values relating to substance use and problems (47%)** **15. Reasons people use (41%).**	**7/8**
**Tang *et al*., 2005**	**China**	**Cross-sectional survey with questionnaire**	**n = 446 physicians from internal medicine, traditional Chinese medicine, psychiatry and surgery.**	**1. Opinions and attitudes towards different aspects of drug use** **2. Familiarity with treatments used in developed countries and opinions about introducing them into China** **3. Training and desired knowledge**	**1. Detoxification-related knowledge (37.9%),** **2. Rehabilitation and relapse prevention knowledge (52.5%),** **3. Other fields of knowledge (aftercare, skill training, etc.) (9.6%)**	**6/8**

## Discussion

The results of this focused review suggest that limited evidence exists on training needs of early career professionals in addiction medicine that could assist the improvement of addiction medicine curricula worldwide. Only three studies were eligible for review. All included studies were cross-sectional study designs using self-reported questionnaire and additional face-to-face interviews (one study). The included studies reported very heterogeneous training needs, including rehabilitation, relapse prevention, buprenorphine induction and risk assessment. While non-random samples and non-validated instruments were used, all studies were deemed to have a low risk of bias, as determined by appraisal using the JBI Critical Appraisal Tool (Moola *et al.*, 2017).

One explanation for the limited findings may be attributable to the relative lack of focus on the early-career education of professionals working in the field of addiction medicine ( The National Center on Addiction and Substance Abuse at Columbia University, 2000; Ayu *et al.*, 2015). In turn, as shown by our focused review, the specific training needs of this population remain under-documented and under-researched. In this respect, developed countries such as North America have new educational pathways that attract growing numbers of health professionals into addiction medicine fellowships. However, in comparison to previous reviews ( El‐Guebaly *et al.*, 2002; Pavlovská *et al.*, 2016), our study identified only one eligible article from North America ( Cunningham *et al.*, 2006), in addition to one from China ( Tang *et al.*, 2005), and another from England ( Galvani and Forrester, 2011). This implies a common lack of structured needs assessments that could inform educational policy in this key stage of training, internationally. In this respect, the WHO Atlas on Substance Use 2017 (ATLAS-SU) study found addiction treatment training programmes lacking at all educational levels internationally ( World Health Organization, 2017b). In addition to insufficient training options in most countries for addiction medicine professionals, a disparity also exists between low and high income countries ( World Health Organization, 2017a).

Furthermore, there appear to be very few valid ways to identify the training needs of ECPAMs. Although the studies were determined to be low risk of bias overall, the training needs summarised in this review were assessed using cross-sectional designs and questionnaires or interviews that were not validated. Evaluation of validated instruments, such as the Training Needs Assessment ( Pinxten *et al.*, 2019), in this population can strengthen the designs of future studies in this evolving body of research. Similar to the WHO studies, previous reviews identified a sharp imbalance in the implementation of established addiction medicine curricula between western and non-western nations ( Ayu *et al.*, 2015), as well as a general lack of training in many areas related to substance use treatment internationally ( El‐Guebaly *et al.*, 2002; Pavlovská *et al.*, 2016). However, compared to the present review, none of them used the structured BEME approach to evaluate ECPAMs, or assessed the risk of bias among the reviewed studies. Applying this approach reduced the number of eligible quality studies on ECPAM training needs. As such, our review identified an important weakness in the quality of education literature that should be addressed in future studies. This review identified nineteen self-assessed training needs. Future reviews should expand the search terminology to include these training needs, in order to examine whether these training needs are also relevant for health care professionals in other career stages of addiction medicine.

Since we have been working with a rigorous review process, according to BEME guidance, the conclusions of our review provide greater insights into the body of evidence on self-assessed needs compared to the previous reviews. More specifically, the review by Ayu *et al*. ( Ayu *et al.*, 2015) identified imbalance in training implementation between low- and high-income countries ( Ayu *et al.*, 2016). In this respect, two overviews by Pavlovska *et al*. ( 2016, 2018) mapped the university-based addiction studies programmes in Europe and the United States ( Pavlovská *et al.*, 2016; Pavlovská *et al.*, 2018). While not specifically focused on a particular country or setting, our review is the first to examine self-assessed training needs of early career professionals working in the field of addiction medicine internationally ( Arya *et al.*, 2019).

This review is limited in several ways. First, there is a very small body of literature regarding the self-assessed training needs of ECPAMs. The small body of literature, and the strict inclusion criteria (i.e., English language studies), contributed to a few included studies. Future reviews should examine training needs of mid-career and established professionals. Second, despite a systematic database search, this study might have missed non-published works or grey-literature studies. However, extensive expert consultations did not indicate any additional literature sources that would influence final results. Third, the narrow focus of the review question, coupled with the risk of bias assessment, resulted in the exclusion of most training evaluations that were included in previous reviews ( El‐Guebaly *et al.*, 2002; Ayu *et al.*, 2015; Pavlovská *et al.*, 2016). Nevertheless, using the BEME guidance, the rigorous review process included two reviewers screening citations for eligibility, rating study quality and extracting data from included studies. In addition, the use of a review allowed for exploration of the underlying central concepts of this area of research that was particularly useful in consideration of the heterogeneity of training needs identified ( Gordon *et al.*, 2019). Finally, with respect to study quality, we identified only small, cross-sectional studies. Given these multiple biases, as well as the heterogeneous outcome reporting, our results are limited for making recommendations for educational programmes but are indicative of a limited scope of the literature on early career addiction medicine education. Future studies should adhere to rigorous protocols, using validated instruments (questionnaire, checklist inspired by existing curricula, etc.) in order to be able to integrate the results in reviews or meta-analyses to inform future educational interventions.

In conclusion, there is little evidence on the training needs found in this focused review. Training needs assessment of early career professionals working in the field of addiction medicine is a priority. As fellowships in addiction medicine emerge internationally, core competencies can also be useful to identify and address educational needs, though the self-reported needs of early career professionals must be systematically integrated.

## Take Home Messages

All included studies surveyed ECPAM using self-reported questionnaires, with one study using face-to-face interviews. Participants included residents, physicians and social workers. All studies had a low risk of bias, and reported a wide range of training needs including rehabilitation, relapse prevention, buprenorphine treatment and risk assessment. There is little evidence for and substantial heterogeneity of training needs of ECPAM found in this review, particularly at the level of skills and knowledge. ECPAM training needs assessments are a priority.

## Notes On Contributors

Jan Klimas, Msc, PhD: Research Fellow at the School of Medicine, University College Dublin, Ireland. (ORCiD: https://orcid.org/0000-0002-5179-0052).

Damien Kelly: Medical Student and summer studentship recipient at the School of Medicine, University College Dublin, Ireland.

Ahmed Adam, MPH: was a Research Assistant on this Focused Review, assisting with systematic searches, study selection and data extraction at the British Columbia Centre on Substance Use.

Sidharth Arya, MBBS, MD: Assistant Professor, State Drug Dependence Treatment Centre, Institute of Mental Health, Pt BDS University of Health Sciences, India.

Blanca Iciar Indave Ruiz, MD, MPH, PhD: Research Assistant, National Center for Epidemiology, Carlos III Institute of Health, Spain.

Dzimtry Krupchanka, MD, MSc, PhD: Medical Officer, Management of Substance Abuse Unit, Department of Mental Health and Substance Abuse, World Health Organization, Geneva, Switzerland. (ORCiD: https://orcid.org/0000-0003-0126-5281).

Michee-Ana Hamilton, MSc: Research Assistant on Systematic Reviews at the British Columbia Centre on Substance Use, Canada.

Thomas Dennehy, MD: General Practitioner and Lecturer/Assistant Professor at the School of Medicine, University College Dublin.

Evan Wood, MD, PhD, ABIM, FRCPC: Professor of Medicine at the University of British Columbia. Chief Executive Director of the British Columbia Centre on Substance Use, Canada.

Walter Cullen, MD, MCGP: General Practitioner and Professor of Urban Health at the School of Medicine, University College Dublin.
