# Supplementary material for: What Are the Self-Assessed Training Needs of Early Career Professionals in Addiction Medicine? A BEME Focused Review
Source: MedEdPublish (2016). 2020 Apr 3;9:62. [Version 1] doi: 10.15694/mep.2020.000062.1 (PMC10697484; doi:10.15694/mep.2020.000062.1)
Supplement: Peer review history

# Reviewer report: Reviewer response for version 1

**DOI:** 10.21956/mep.19996.r30456

**Published:** 2020-04-03

**Authors:** Trudie Roberts

**Affiliations:** 1 Leeds Institute of Medical Education

This review has been migrated. The reviewer awarded 5 stars out of 5 I was surprised at the findings of this paper. As the authors point out this area is a huge health and economic burden for many countries. I would suggest that not only is there a need for more targeted training for ECPAM but also for all trainees who are dealing with frontline patient encounters and that possibly some of the targeted training that needs to be developed could be repurposed for more general use. Thank you for highlighting this area of serious omission you next mission (should you choose to accept it) is to rectify this deficiency.

Reviewer Expertise:

NA

No decision status is available

# Reviewer report: Reviewer response for version 1

**DOI:** 10.21956/mep.19996.r30455

**Published:** 2020-04-03

**Authors:** Trevor Gibbs

**Affiliations:** 1 AMEE

This review has been migrated. The reviewer awarded 4 stars out of 5 I enjoyed reading this targeted review and like my co-reviewer was very surprised at the lack of structured training in this area, although I think it reflects a similar lack in undergraduate education; I wonder why graduates move into this area. I feel that there are some specialties that address this need, frequently hidden in other curricula, an example would be in GP training programmes.I look to how the authors and perhaps the readers of this review intend to fill that gap

Reviewer Expertise:

NA

No decision status is available
